# Berberine-containing natural-medicine with boiled peanut-OIT induces sustained peanut-tolerance associated with distinct microbiota signature

**DOI:** 10.3389/fimmu.2023.1174907

**Published:** 2023-07-26

**Authors:** Kamal Srivastava, Mingzhuo Cao, Ozkan Fidan, Yanmei Shi, Nan Yang, Anna Nowak-Wegrzyn, Mingsan Miao, Jixun Zhan, Hugh A. Sampson, Xiu-Min Li

**Affiliations:** ^1^ General Nutraceutical Technology, Elmsford, NY, United States; ^2^ Department of Pathology, Microbiology and Immunology, New York Medical College, Valhalla, NY, United States; ^3^ Academy of Chinese Medical Sciences, Henan University of Chinese Medicine, Zhengzhou, China; ^4^ Department of Biological Engineering, Utah State University, Logan, UT, United States; ^5^ Department of Bioengineering, Abdullah Gul University, Kayseri, Türkiye; ^6^ Hassenfeld Children’s Hospital, Department of Pediatrics, New York University (NYU) Grossman School of Medicine, New York, NY, United States; ^7^ Department of Pediatrics, Gastroenterology and Nutrition, Collegium Medicum, University of Warmia and Mazury, Olsztyn, Poland; ^8^ Department of Pediatrics, Icahn School of Medicine at Mount Sinai, New York, NY, United States; ^9^ Department of Otolaryngology, New York Medical College, Valhalla, NY, United States

**Keywords:** peanut allergy, IgE, berberine, microbiota, 16S rDNA, oral immunotherapy (OIT) *Angelica sinensis*

## Abstract

**Background:**

Gut microbiota influence food allergy. We showed that the natural compound berberine reduces IgE and others reported that BBR alters gut microbiota implying a potential role for microbiota changes in BBR function.

**Objective:**

We sought to evaluate an oral Berberine-containing natural medicine with a boiled peanut oral immunotherapy (BNP) regimen as a treatment for food allergy using a murine model and to explore the correlation of treatment-induced changes in gut microbiota with therapeutic outcomes.

**Methods:**

Peanut-allergic (PA) mice, orally sensitized with roasted peanut and cholera toxin, received oral BNP or control treatments. PA mice received periodic post-therapy roasted peanut exposures. Anaphylaxis was assessed by visualization of symptoms and measurement of body temperature. Histamine and serum peanut-specific IgE levels were measured by ELISA. Splenic IgE^+^B cells were assessed by flow cytometry. Fecal pellets were used for sequencing of bacterial 16S rDNA by Illumina MiSeq. Sequencing data were analyzed using built-in analysis platforms.

**Results:**

BNP treatment regimen induced long-term tolerance to peanut accompanied by profound and sustained reduction of IgE, symptom scores, plasma histamine, body temperature, and number of IgE^+^ B cells (*p <*0.001 vs Sham for all). Significant differences were observed for *Firmicutes*/*Bacteroidetes* ratio across treatment groups. Bacterial genera positively correlated with post-challenge histamine and PN-IgE included *Lachnospiraceae*, *Ruminococcaceae*, and *Hydrogenanaerobacterium* (all *Firmicutes*) while *Verrucromicrobiacea*. *Caproiciproducens*, *Enterobacteriaceae*, and *Bacteroidales* were negatively correlated.

**Conclusions:**

BNP is a promising regimen for food allergy treatment and its benefits in a murine model are associated with a distinct microbiota signature.

## 1 Introduction

An appreciation for the role of gut microbiota to modulate immune responses (1, 2) has led to intense interest in the relationship between gut microbiota and food allergy (3–5), which continues to be a major health problem worldwide. Food allergy treatments have been elusive and the development of lasting cures for food allergy remains an active area of research (6–9). Several studies in human and animal models of food allergy have demonstrated an association with distinct gut microbiota profiles (3, 10–12). In addition to microbiota characteristics of food-allergic patients, several reports have described changes in gut microbiota that accompany natural resolution (4, 13) or treatment-induced improvement in disease (14–17), suggesting a role for gut microbes in the outcome of food allergy treatment. We have previously demonstrated that botanical medicines derived from Traditional Chinese Medicine provide persistent protection from anaphylaxis and cause long-term reduction of IgE and beneficial re-programming of the T-helper cytokine profile in mice with peanut allergy (18, 19), concomitant peanut/tree nut allergy (20), and multiple food allergies (21). Building upon these studies, we showed that berberine (BBR), a quinolizidine alkaloid present in *Phellodendron chinensis* has the remarkable ability to suppress IgE production in IgE-producing human myeloma cells and peripheral blood mononuclear cells obtained from allergic patients (22). *In vivo* validation of this property and the evaluation of its potential for food allergy treatment has not been possible due to very poor BBR biovailability (23, 24). Using *in vitro* approaches, we observed that BBR bioavailability was enhanced after oral feeding of food allergy herbal formulas FAHF-2 and B-FAHF-2. Subsequent profiling of the individual constituent herbs of FAHF-2 for the ability to enhance BBR uptake by CACO-2 cells led to the identification of *Angelica sinensis* (AS) as one of the component herbs with the ability to increase BBR uptake (25). Roasted peanut powder (flour) oral immunotherapy (OIT) is a current FDA-approved clinical treatment for PN allergy, but it has the potential for side effects, such as gastrointestinal inflammation and immediate allergic symptoms, including anaphylaxis (26–29). Boiled PN has been shown to be less reactive than roasted PN by us and others (30–32). Thus, in the current study, we tested a combined therapy regimen using BBR, water extracts of AS and boiled PN OIT referred to together as BBR-containing Natural-medicine with boiled Peanut immunotherapy (BNP) (33). In addition to investigating the effects of the treatments on allergic responses, we explored whether the BNP regimen would have consequences on the gut microbiota profile and whether observed changes correlated with disease status.

## 2 Materials and methods

### 2.1 Peanut sensitization and challenge and treatment

#### 2.1.1 Peanut sensitization and challenge of mice

Five-week-old female C3H/HeJ mice (PA mice) purchased from the Jackson Laboratory (Bar Harbor, ME) were maintained in pathogen-free facilities at the Mount Sinai vivarium according to standard guidelines for the care and use of animals (34).

**Figure 1:**
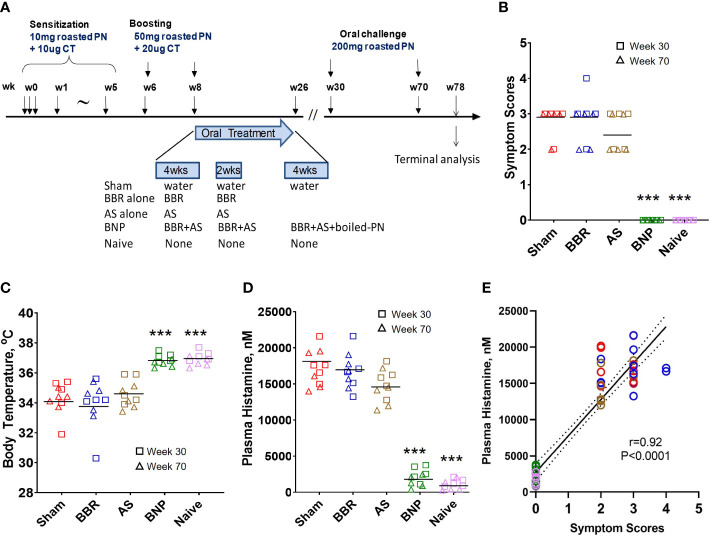
Experimental design and anaphylactic reactions at challenge. **(A)** Experimental protocol. *In vivo*, experimental protocol- 6-week-old female C3H/HeJ mice were subjected to oral peanut sensitization in the presence of cholera toxin from week 0 through week 5 and boosted thereafter at week 6 and week 8. Mice were then given BNP therapy between W8-W26 as described in methods. Mice were challenged at W30 (Data shown using square symbols) and W70 (Data shown using triangle symbols). The experiment was terminated at W78. **(B)** Symptom scores were assigned 30 minutes after challenge using criteria described in methods. **(C)** Body temperature was measured by rectal probe after assessment of symptom scores. **(D)** Plasma histamine levels were measured by duplicate ELISA of individual plasma samples harvested from blood collected 30 minutes after the measurement of body temperatures. **(E)** Analysis of correlation between symptom scores and plasma histamine at W30 and W70 challenge time points. Color key for symbols: Red-Sham, Blue-BBR, Brown-AS, Green-BNP, Pink-Naïve. Bars in B are group medians and in C and D are group means. In E solid line represents the regression line and the dashed line represents the 95% confidence interval. Red color represents sham, blue color represents BBR, tan color represents AS, green color represents BNP, and purple color represents naïve. N=5 mice/group. Data represent 10 readouts from a combination of W30 (square symbols) and W70 (triangle symbols) challenges. ***p<0.001 vs Sham.

C3H/HeJ mice were orally sensitized with roasted PN and cholera toxin as previously published by our research group (35, 36). As shown in **Figure 1A**, a detailed experimental protocol was established as previously reported with minor modifications (37). Mice were intragastrically (i.g.) sensitized with 10 mg of homogenized roasted peanut in a 0.5 ml PBS containing 75 mg sodium bicarbonate, 10 µg of the mucosal adjuvant cholera toxin (List Laboratories, CA), and 16.5 µl (1.1 µl/g body weight) of 80 proof Stolichnaya Vodka^®^ (a source of food grade ethanol) to neutralize stomach pH and to increase gastrointestinal permeability. A boosting dose of 50 mg PN was given at weeks 6 and 8 using the same gavage solution as above. Naïve mice were not sensitized. Oral challenges with 200 mg roasted PN were given at weeks 30 and 50.

#### 2.1.2 Treatment regimen

The course of oral treatment was started at week 8 after completion of the sensitization and boosting protocol and continued until week 26. This represents a therapeutic protocol as we have shown that mice developed peanut IgE sensitization and exhibited reactions in response to peanut challenge at week 8 (20, 38). During the 18-week treatment regimen, PN allergic mice were orally treated with a combination of daily BBR and AS extract in three courses. Two off-treatment intervals of 2 and 4 weeks were included to assess the potential for off-therapy IgE rebound. Boiled peanut OIT was added for the final 4 weeks of the BBR+AS therapy, altogether referred to as BNP. Control groups included PA mice given either 2 mg BBR/day (Sigma-Aldrich), AS water extract (10 mg/day), sham-treated PA mice, and naïve mice. Water extract of AS was prepared by Sanmenxia Shanshui Fangzheng Biotechnology Ltd, Sanmenxia, China as follows. Verified AS raw herb was cut to obtain 1-2 cm pieces and soaked in excess water (1:10 v/v) overnight. The water/AS mixture was then boiled for 2 hours. The aqueous phase was collected through a filtration process and the residue was subjected to a second round of hot water extraction (1:8 v/v). The two decoctions were collected and concentrated to a density of 1.15-1.20 g/ml. The condensed extract was then dried at 65 °C under vacuum. Dried AS extract was subsequently ground to a fine powder for further use. The product quality control was conducted by HPLC fingerprint. BBR was purchased from Sigma-Aldrich and the ratio of BBR: AS was determined by previous *in vitro* uptake studies and estimation of BBR in the daily dose of 12 mg B-FAHF-2 for mice (25). Raw peanuts without shells but with skin were boiled in water for 30 minutes and subsequently homogenized in phosphate-buffered saline. Daily boiled peanut OIT was given at a dose of 10 mg/day. All treatments were given by oral gavage dissolved in drinking water at a volume of 0.5ml/gavage.

### 2.2 Assessment of hypersensitivity reactions

Anaphylactic symptoms were evaluated 30-40 minutes after oral PN challenge as described previously (37). The severity of observed symptoms on a scale of 0 (no reactions)-5 (death due to anaphylaxis was scored utilizing the scoring system described previously (37). 0 - no symptoms; 1 - scratching and rubbing around the snout and head; 2 - puffiness around the eyes and snout, pilar erecti, reduced activity, and/or decreased activity with increased respiratory rate; 3 - wheezing, labored respiration, cyanosis around the mouth and the tail; 4 - no activity after prodding, or tremor and convulsion; 5 - death. Cage identities were concealed during the visual assessment of anaphylactic symptoms. Rectal temperatures were measured using a rectal probe (Harvard Apparatus, NJ, USA).

### 2.3 Measurement of plasma histamine levels and PN-specific IgE

ELISA measurements for plasma histamine levels and PN-specific IgE have been detailed as described previously, plasma was harvested within 20 minutes after blood collection at challenge and stored at -80 °C until used (20, 37). Histamine was measured using an enzyme immunoassay kit (Fisher Scientific, NJ) as described by the manufacturer. Peanut-specific IgE levels in serum were measured as reported previously (20). Briefly, microtiter plates were coated with crude peanut extract (CPE) (39) or standard of purified anti-mouse IgE (BDBiosciences, CA, USA) and held overnight at 4 °C. After washing, plates were blocked with 2% Bovine Serum Albumin-PBS. Washed plates were incubated with samples overnight at 4 °C and developed using biotinylated anti-IgE detection antibodies (BD Biosciences, CA, USA), avidin-peroxidase, and ABTS substrate (KPL, MN, USA).

### 2.4 Flow cytometry analysis

A single-cell suspension of splenocytes was suspended in ice-cold staining buffer (PBS including 0.5 mM EDTA, 0.05 mM Sodium Azide, and 0.5% BSA). First, surface staining was performed by incubating cells with unlabeled anti-IgE (to block membrane IgE), BV605 anti-B220, BV711-anti-CD3, anti-CD16/32 (Fc-block), all from BD Biosciences, CA). Live-dead discriminating dye (Live-Dead Aqua, Invitrogen, CA) was also included at this point. Cells were incubated in the dark for 30 minutes on ice. Cells were then washed 3 times with a staining buffer. Cells were then incubated with fixation/permeabilization buffer for 15 minutes. Cells were washed with permeabilization buffer and incubated with FITC-anti IgE, in permeabilization buffer for 30 minutes in the dark on ice. Cells were then again washed 3 times with a staining buffer. Cells were treated with Cytofix buffer for 15 minutes for post-fixation. Then they were resuspended in 200 µl staining buffer for cell acquisition on an LSRII flow cytometer (Becton Dickinson, CA). Flow cytometry analysis was performed using Flow jo (Tree Star) as follows. Live cells were selected by excluding Live-Dead Aqua-positive cells. Of live cells, singlet staining was selected on the basis of FSC-A/FSC-H profile. Singlet cells were then analyzed for IgE+ B cells (FITC-IgE +; BV605-B220+ cells).

### 2.5 Fecal microbiota analysis

Approximately 7-10 fecal pellets from individual mice were collected prior to terminal analyses at week 52 post-therapy. Fecal pellets were aseptically collected and stored at -20 °C. Samples were shipped to Utah State University where sample processing and microbiota studies were performed. Briefly, isolated total community DNA was isolated from the fecal samples using a QIAGen QIAamp DNA stool mini kit according to the instructions provided by the manufacturer. Upon obtaining total DNAs, samples were sent to Idaho State University (ISU) Molecular Core Facility for Illumina MiSeq next-generation sequencing (https://www.isu.edu/research/centers-and-institutes/molecular-research-core-facility/services/). Briefly, first-stage PCR was performed to amplify the V3-V4 region of 16S rRNA from total DNA. Subsequently, the PCR products for each sample were cleaned using Ampure XP beads. Then, a second-stage PCR was performed for Illumina indexing. Upon running Illumina MiSeq for the samples, the raw fastaq files were hosted at the site managed by Idaho State University and their sequencing facility to perform the bioinformatics analysis using Mothur software package and we received processed data files. These files were uploaded to microbiomeanalyst.ca for selected analyses using Mothur output files and SILVA taxonomy as the reference 16S rDNA database.

### 2.6 Statistical analysis

Data were analyzed using GraphPad prism 8 software. Symptom scores, plasma histamine, body temperature, and IgE data were analyzed by One-Way ANOVA on ranks followed by Kruskal Wallis post-test for symptom scores and One-Way ANOVA followed by Dunnett’s post-test for histamine, temperature, and IgE data. Secondary statistical analyses of microbiota data were performed through Welches ANOVA and multiple T-tests using the Holmes-Sidak method to correct for multiple comparisons, generate Spearman R values and graphs of linear regression, and stacked bar and donut charts of microbiota data. *p* values < 0.05 were considered significant.

## 3 Results

### 3.1 Oral BNP treatment confers persistent protection from anaphylactic reactions to oral peanut challenge

As described in **Figure 1A**, oral roasted peanut challenges were given at weeks 30 and 70 of the experimental protocol. At each challenge, mice were evaluated for anaphylactic symptoms, drop in body temperature, and plasma histamine levels. Mice given BNP treatment were completely protected from anaphylaxis as no mouse at either challenge displayed symptoms of anaphylaxis following oral challenge (**Figure 1B**). Groups given BBR alone or AS alone were similar to mice in the Sham group with respect to median symptom scores. Protection from systemic anaphylaxis in the BNP treatment group was also evidenced by a lack of temperature drop after peanut challenges (**Figure 1C**). Mean body temperature was similar to naïve mice and significantly higher than mice in the Sham group (*p*<0.001 vs Sham). Plasma histamine levels in mice in the BNP mice were significantly lower than those observed for Sham mice (*p*<0.001 vs Sham, **Figure 1D**). Aggregate evaluation of plasma histamine levels of all mice and correlation with symptom scores (r=0.92, *p*<0.0001, **Figure 1E**) was found to be of robust strength and statistical significance underscoring the central role of histamine with anaphylactic severity in our model and its suitability as a marker of anaphylaxis used for further analysis in our study.

### 3.2 BNP-treated mice show rapid, profound, and sustained decline of PN-specific IgE that was accompanied by reduction of IgE^+^ B cells

**Figure 2:**
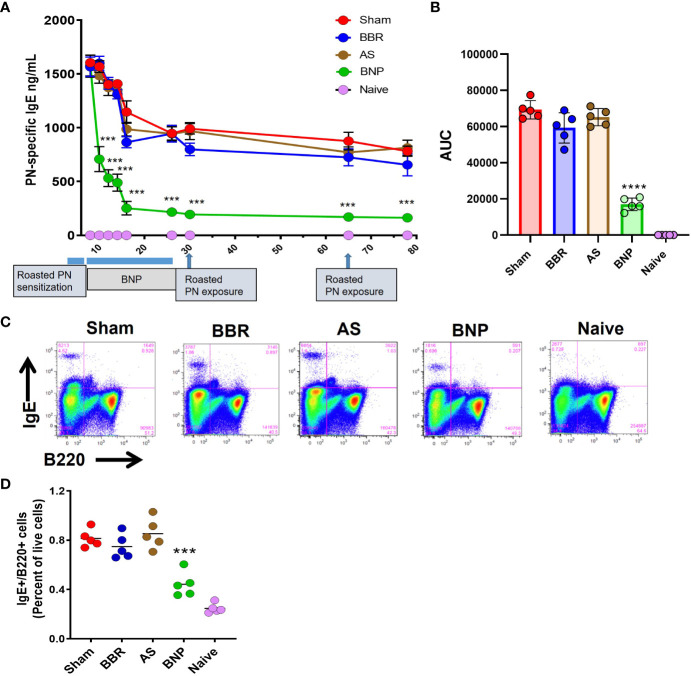
Peanut-specific IgE and IgE^+^ B cells. **(A)** Peanut-specific IgE levels measured by duplicate ELISA of individual samples. **(B)** Differences in PN-specific IgE expressed as AUC values. **(C)** Flow cytometry panels showing percentages of IgE^+^ B cells in the spleen of representative mice for each group. Splenocytes from individual mice were processed for flow cytometry staining to evaluate IgE^+^ B cells using FITC-IgE and BV605-B220 antibodies **(D)** Scatter graph of data for the percentage of IgE^+^ B cells in each group. Data in A shown as group Means± SEM. Bars in B and C are group Means. N=5 mice/group. ***, **** represent *p*<0.001, 0.0001 vs Sham.

Within 2-4 weeks after commencing treatment, PN-specific IgE levels were observed to decline, as shown in **Figure 2A**. Reduction in PN-specific IgE was rapid, achieving nearly 70% reduction within 2 weeks of starting treatment. PN-specific IgE was reduced by nearly 80% in the BNP group by the end of treatment at week 26. Importantly no significant increases in PN-specific IgE were observed with the introduction of boiled peanut OIT during the final 4 weeks of BNP treatment and no symptoms were observed. Reduction in peanut-specific IgE was sustained over the remainder of the protocol post-therapy, which included oral roasted peanut challenges at weeks 30 and 70. Overall, as shown by the comparison of AUC values in **Figure 2B**, PN-specific IgE was markedly reduced in BNP-treated mice (*p*<0.0001). Groups given BBR alone or AS alone showed no significant reduction of IgE over the course of the experiment compared with the sham-treated group. Mice were sacrificed at week 78 (52 weeks after stopping therapy) for terminal analyses. At this time IgE^+^ B (IgE^+^B220^+^) cells were evaluated in spleens of mice using flow cytometry. BNP-treated mice showed significantly reduced percentages of IgE^+^ B cells and IgE-plasma cells (**Figures 2C, D**, *p*<0.01-0.001 vs Sham). Taken together treatment of peanut-allergic mice with the BNP led to profound and sustained reduction of peanut-specific IgE and IgE^+^B cells.

### 3.3 Gut microbiota of allergic mice given BNP significantly differs from sham allergic mice and is more similar to naïve controls

In light of recent reports that demonstrate the association of gut microbiota signatures with food allergy in humans (4) and mice (16, 17, 40), we investigated the impact of different treatment groups evaluated in this study for an effective food allergy treatment on the gut microbiota in mice. We performed 16S rDNA sequencing analysis of fecal pellets collected at the end of the study. Overall, 469 operational taxonomic units (OTUs) were identified in the samples across all groups.

**Figure 3:**
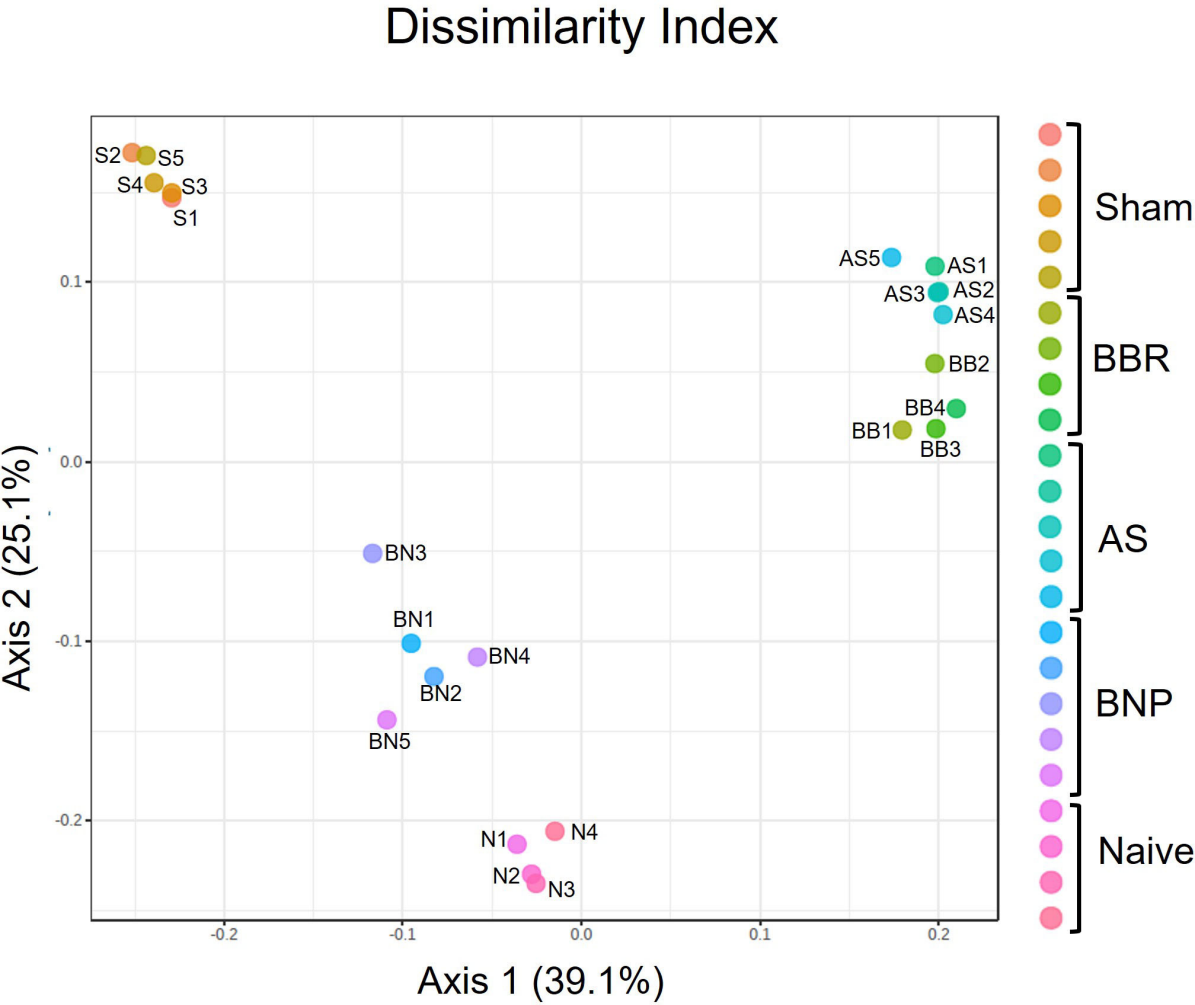
Principle Coordinate analysis of OTUs generated using PERMANOVA. N=4-5 mice/group. Symbols on the graph are as follows: S-Sham, BB-BBR, BN-BNP, AS-AS, and N-Naïve.

Principal Coordinate Analysis (PCoA) at the OTU level (**Figure 3**) indicated a closer relation between OTU profiles of mice treated with BNP and Naïve control mice, than with Sham mice or mice given BBR alone or AS alone. However, the richness of the microbiota at the OTU level was not different across experimental groups (data not shown). Together these data suggest that experimental groups in our study come to acquire distinct microbiota signatures and that mice given the BNP treatment regimen display a microbiota profile that is more closely related to naïve mice.

### 3.4 Phylum composition of gut microbiota in mice treated with the BNP regimen shows a lower *Firmicutes*/*Bacteroidetes* ratio and a higher abundance of phyla associated with beneficial metabolic status

**Figure 4:**
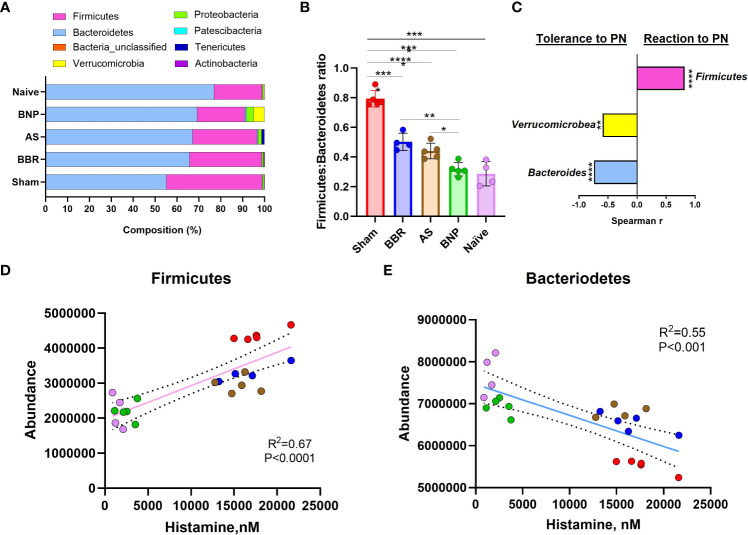
Phylum Abundance data. **(A)**. Actual Phylum abundance values obtained using Mothur outputs were used to generate phylum composition data. **(B)**. *Firmicutes : Bacteroidetes* ratio. **(C)**. Spearman Correlation Index (r) using GraphPad Prism. **(D)** & **(E)** are linear regression plots for phylum abundance of *Firmicutes* and *Bacteroidetes* respectively against Plasma histamine levels at week 70 challenge. Color key for symbols in D & E: Red-Sham, Blue-BBR, Brown-AS, Green-BNP, Pink-Naive. N=4-5 mice/group *p<0.05; **p<0.01; ****p<0.0001 vs Sham.

Evaluation of actual abundance at the phylum level showed that a major portion of the gut microbiome in all experimental groups in our study was accounted for by the *Firmicutes* and *Bacteroidetes* (**Figure 4A**). A high *Firmicutes*/*Bacteroidetes* ratio is reported to be correlated with inflammation, metabolic dysregulation, and autism spectrum accompanied by gastric disturbances (41–43). In our study, the Sham group displayed higher *Firmicutes* to *Bacteroidetes* ratio compared to the Naïve group (*p*<0.0001, **Figure 4B**). Mice in the BNP treatment group had lower *Firmicutes : Bacteroidetes* ratios compared to all other groups except naïve mice (*p*<0.0001-0.05, **Figure 4B**). Interestingly, *Verrucromicrobiacea* was noticeably increased in the group given BNP (**Figure 4A**). *Verrucromicrobiacea* has been reported to be associated with improved metabolic status in humans and mice. Its abundance is also correlated with the integrity of the intestinal epithelium. To understand the relationship between observed phylum and disease status in our model, we evaluated correlations between phylum abundances and levels of plasma histamine at final challenge. Histamine is a central mediator of anaphylactic responses, and the histamine level is strongly correlated with the severity of symptoms in this model, (**Figure 1D**) as demonstrated in our previous studies. We found that plasma histamine levels were strongly correlated with *Firmicutes* abundance (**Figures 4C, D**) and strongly but inversely correlated with *Bacteroidetes* (**Figures 4C, E**). Inverse correlations with histamine, albeit of moderate strength were also observed for *Verricumicrobia* (**Figure 4C**). These results suggested that enhancement of the phyla *Bacteroidetes* and *Verrucomicrobia* might be linked to the induction of peanut tolerance by BNP treatment.

### 3.5 *Bacteroidales*, *Tannerellacea*, and *Clostridale* Family XIII_ge were found to be negatively correlated with plasma histamine and IgE

**Figure 5:**
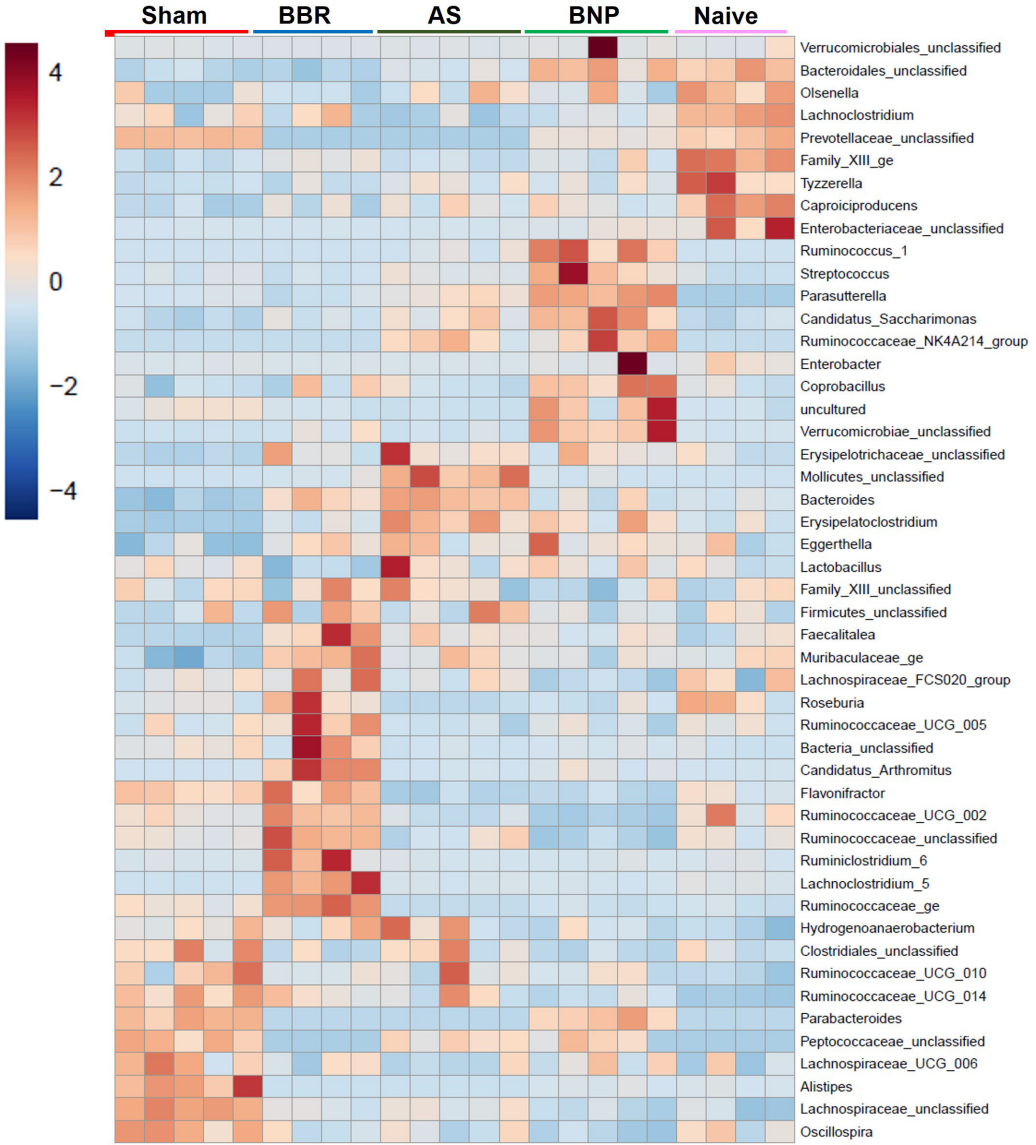
Genus Heat map. Heat map generated by Mothur output of genus abundance data using the built-in method at microbiomeanalyst.edu.ca.

A more in-depth understanding of microbiota perturbations in experimental groups was possible by evaluation of taxa abundance at the genus level. As shown in the heat map in **Figure 5**, several regions of the heat map show taxa enrichment profiles unique to the experimental groups in our study. *Ruminococcaceae_UGC_014* and *Peptococcacea* showed highly differential enrichment profiles between Sham and Naïve groups with high enrichment in Sham and lower in Naïve mice. In contrast, *Bacteroidales_unclassified* were most enriched in Naïve and BNP groups relative to Sham. *Bacteroides* and *Erysepaloclostridium* were the most decreased in the Sham group. *Verrucomicrobiae_unclassified* were only enriched in the BNP group.

**Figure 6:**
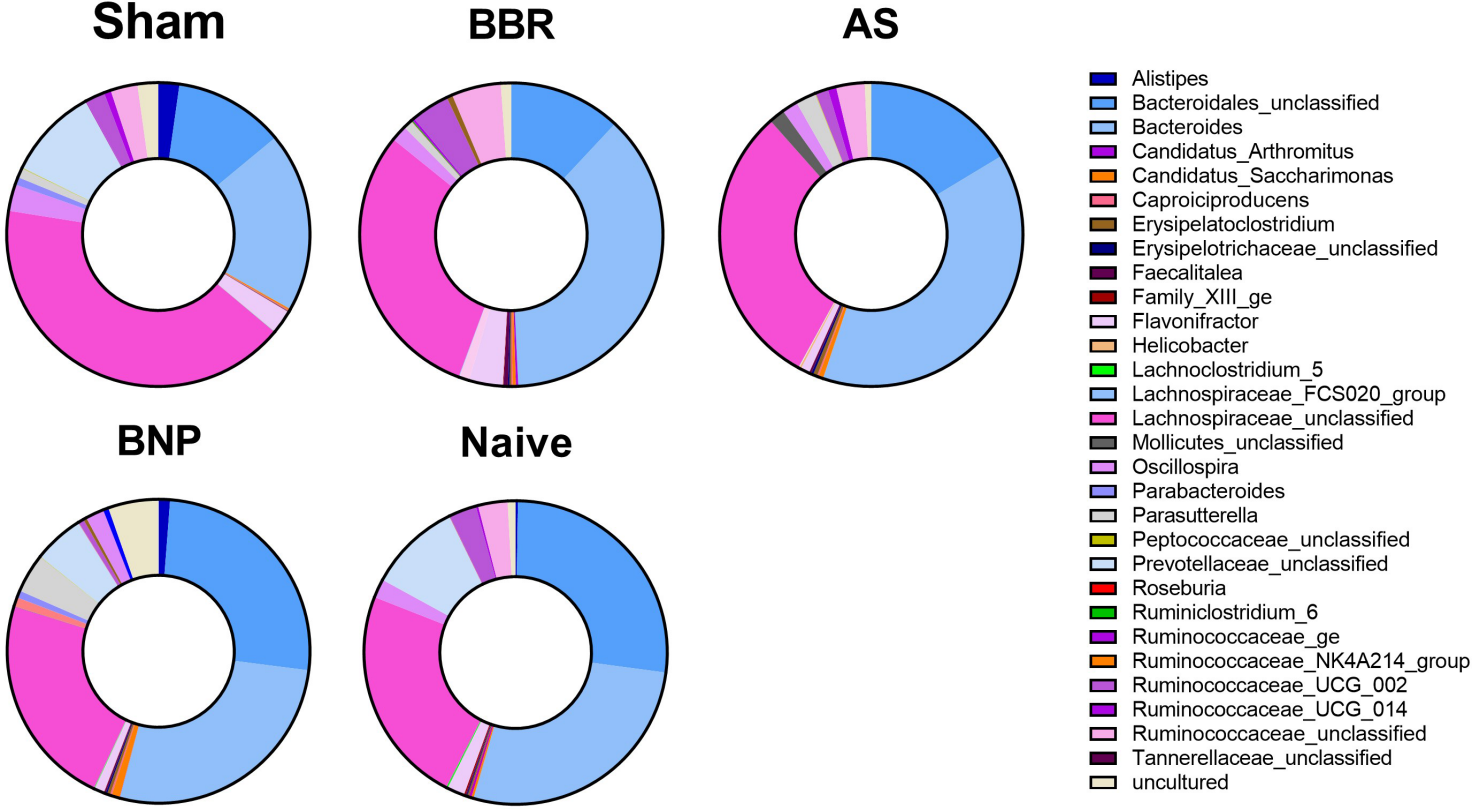
Composition of taxa with significantly different abundance levels compared to the Sham group. Donut plots of actual genus abundance data for taxa that were statistically significant (*p*<0.05) for any experimental group vs Sham using Welch’s ANOVA. Taxa belonging to *Firmicutes* are shown in shades of pink and those belonging to *Bacteroidetes* are indicated in shades of blue. N=4-5 mice/group.

We then evaluated the differential composition of those bacterial genera which had abundances that were significantly different from the sham group and at least one of the other experimental groups (**Figure 6**) as assessed by Welch’s ANOVA (P value thresh hold set to <0.05). The majority of taxa belonged either to *Firmicute*s (indicated in shades of pink) or *Bacteroidetes* (indicated in shades of blue). The greatest changes were observed for the *Bacteroides* and *Bacteroidales_unclassified*, which were increased in BNP and Naïve groups compared to the Sham group. Specifically, *Bacteroides_unclassified* representation in Naïve and BNP groups were essentially similar and increased compared to all other experimental groups. *Lachnospiraceae_unclassified* (a *Firmicutes* member) were more abundant in the Sham, BBR, and AS groups relative to Naïve and BNP mice.

**Figure 7:**
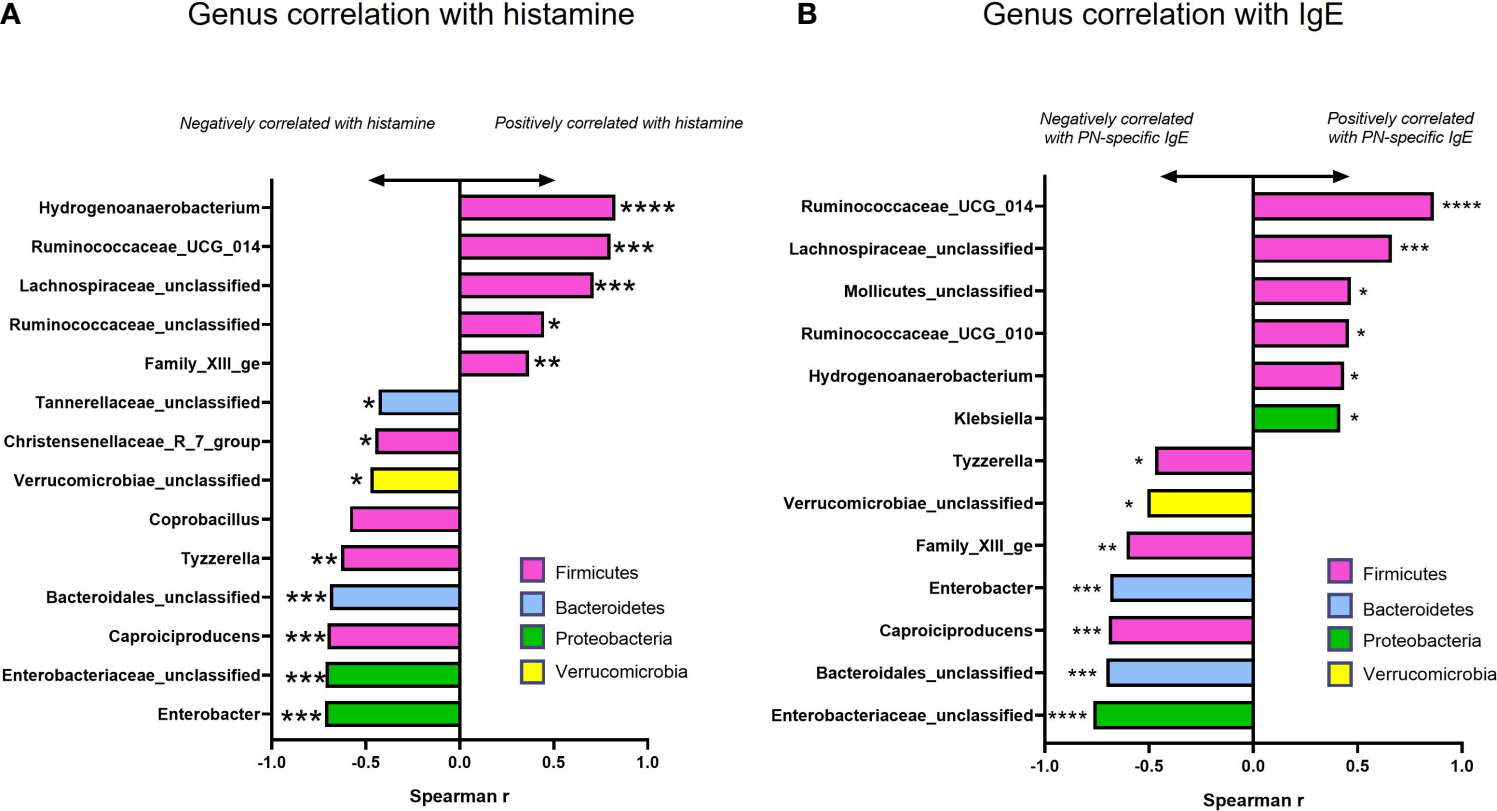
Correlation and abundance data among taxa with significantly different abundance values compared to Sham. Spearman Correlation Index (r) for actual genus abundances for taxa with statistically different abundance compared to Sham against plasma histamine levels **(A)** and IgE **(B)** at week 70 challenge using GraphPad Prism. Color of bars indicates Phylum assignment. Color key: Pink-*Firmicutes*, Blue-*Bacteroidetes*, Green-*Proteobacteria*, Yellow-*Verrucomicrobia*. *p<0.05; **p<0.01; ***p<0.001; ****p<0.0001 vs Sham.

**Figure 8:**
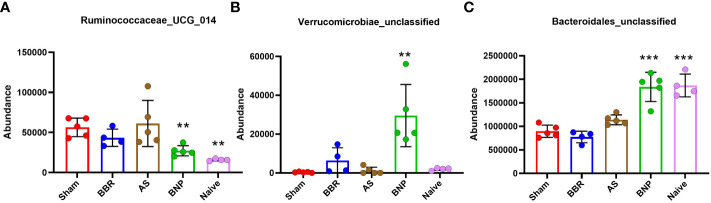
Actual abundance values of 3 bacterial genera (**A** Ruminococcaceae_UCG_014, **B** Verrucomicrobiae_unclassified, **C** Bacteroidales_unclassified) with positive or negative correlation with plasma histamine and IgE at week 70 challenge that were also significantly different from Sham. N=4-5 mice/group. **p<0.01; ***p<0.001 vs Sham.

To determine whether changes in the abundance of specific bacterial genera were associated with treatment benefits, we evaluated the correlation of genera in **Figure 6** (among those with significantly different abundance compared to sham) with plasma histamine and IgE levels at the time of final challenge. We also noted the phylum of each candidate (*Firmicutes*-Pink, *Bacteroidetes-* Blue, *Proteobacteria*- Green, and *Verrucomicrobia*- Yellow). Results of these analyses (**Figure 7**) showed that *Caproiciproducens* (Firmicutes), Family XIII_ge (Firmicutes), *Tannerellaceae* (*Bateroidetes*), and *Bacteroidales unclassified* (*Bacteroidetes*) had a strong inverse correlation with plasma histamine (**Figure 7A**) and IgE (**Figure 7B**) suggesting that these taxa may be associated with protection. Conversely, *Firmicutes* members *Lachnospiraceae_unclassified* and *Ruminococcaceae_UGC_014* were strongly and positively associated with these disease markers implying a possible role in food allergy pathology. *Verrucomicrobia* showed a moderate but significant inverse correlation with both IgE and histamine. Statistically significant differences in actual abundance values between Sham and BNP groups were found for *Ruminococcaceae_UGC_014*, (**Figure 8A**) which were reduced in the BNP group, and *Verrucomicrobia* and *Bacteroidales* which were significantly increased (**Figures 8B, C**). Further research to identify specific bacterial species and transfer experiments to prove causation are needed to validate the functionality of these findings.

## 4 Discussion

The findings of our current study showed that the design of a food allergy treatment regimen containing the medicinal natural compound BBR is efficacious in a murine model of peanut allergy and is associated with a distinct gut microbiota signature. Our observation that BNP is therapeutically equivalent to and with regard to IgE reduction, even superior to parent TCM formulas FAHF-2 and E-B-FAHF-2 represents (Yang et al. Manuscript in preparation) a major achievement in our research efforts to develop an orally available BBR-centered food allergy treatment. We were the first to identify the IgE-lowering abilities of BBR *in vitro (* 22), but poor bioavailability was an obstacle to its *in vivo* application as a potential medicine to treat food allergies. Research efforts to solve this problem led us to the identification of AS, one of the component herbs of FAHF-2 and B-FAHF-2, which we found to enhance BBR uptake in CACO-2 cells (25). Our current data support these earlier findings and shows that BBR-uptake-enhancing natural medicine AS (*Angelica* species) is necessary for the translation of the IgE- lowering effect of BBR *in vivo*. AS have been shown to contain natural inhibitors of p-glycoprotein (p-Gp) (44–46). Since p-Gp has been shown to promote intestinal efflux of BBR driving down its uptake (47–49), potential inhibition by AS likely enhances BBR bioavailability in our system. BBR has been used in Traditional Chinese Medicine as a treatment for diarrhea (50) and more recently it has been used as medicine for diabetes (51, 52), metabolic syndrome (52, 53), and hyperlipidemia (54, 55). As a result, there is extensive interest in BBR-modulation of gut microbiota as these diseases are intimately linked to gut microbiota responses to diet. Several publications have reported alteration of the gut microbiota by BBR in various disease models and humans (56–59). In light of this and the growing appreciation for the role of gut microbiota in food allergy, we were interested in exploring potential alterations in gut microbiota in our food allergy model and the relationship of these changes to the therapeutic benefits of BNP.

Analysis of 16S rDNA sequences in fecal pellets obtained from mice at the time of final peanut exposure in our study showed that PA mice had higher a *Firmicutes*/*Bacteroidetes* ratio than Naïve mice, although microbiota richness at the OTU level was not significantly different. Mice treated with BNP regimens were more similar to Naïve mice in this regard. A lower *Firmicutes*/*Bacteroidetes* ratio is generally associated with healthier metabolic status and a high *Firmicutes*/*Bacteroidetes* ratio is observed in obesity, autistic children with gastric disturbances (41) (60), and in murine models of allergic asthma (61, 62) whereas a higher *Firmicutes*/*Bacteroidetes* ratio is considered beneficial in the setting of autoimmune inflammatory conditions such as colitis and IBD (63–65). No strong evidence has been reported for the impact of *Firmicutes*/*Bacteroidetes* ratio on food allergy. Using plasma histamine levels at challenge, a biomarker of food allergy reactions, for correlation analysis, we found that *Firmicutes* abundance was strongly and positively correlated with histamine levels at challenge whereas *Bacteroidetes* had a strong negative correlation. The negative association of *Bacteroidetes* abundance with allergic reactions in our murine study was in line with findings in humans as reviewed by Shu et al. (66), where the lower levels of *Bacteroides* subsequent to maternal intrapartum antibiotic exposure were implicated in higher sensitization rates in cesarean born children.

Although at the Phylum level, *Firmicutes* abundance was positively correlated with allergy in our study, deeper analyses revealed that at the genus level, *Firmicutes* members exhibited both positive and negative correlations with histamine and PN-IgE levels. This is consistent with previous reports of *Firmicutes* members such as certain *Clostridiales* to be beneficial in the context of food allergy (13, 67).

In our study, genus-level analysis revealed that some *Firmicutes* members such as *Lachnospiraceae_unclassified* and *Ruminococcaceae_UGC_014* were strongly and positively correlated with histamine levels and IgE. Enrichment for *Lachnospiraceae* and *Ruminococcacea* members has been reported in patients with cow’s milk and egg allergy and in murine models of peanut allergy (11, 14, 15). In contrast, the *Firmicutes* member *Caproiciproducens*, belonging to the order *Clostridiales* had robust negative correlations with histamine and IgE. Other taxa negatively associated with histamine levels and IgE were *Bacteroidales* and *Verrucomicrobia*. *Bacteroidales* have been associated with improved integrity of the intestinal epithelium and *Verrucomicrobia* phylum has been shown to be associated with restoration of metabolic health (68, 69). Knowledge about gut-inhabiting *Verrucumicrobia* appears to be limited to *Akkermansia muciniphila (* 70, 71). *A. muciniphila*, a mucus-degrading gut bacterium has recently received much attention due to its association with improved metabolic health (72), anti-inflammatory profile (73, 74) and ability to promote the integrity of the intestinal epithelium (69, 75, 76). More research is needed to definitively understand whether a BNP-induced shift in microbiota drives an early and rapid decline of IgE or whether these changes are more indicative of a gut microbial community reprogramming as a result of BBR-induced reduction of allergic responses. Comparing microbiota findings in our study to other food allergy studies described previously has proven to be difficult due to published studies being drawn from varying settings of food allergy. Gut microbiota studies in allergy need to be controlled for several aspects such as patient age, IgE vs non-IgE, and specific foods. Murine model data also is drawn from models using various strains, antigens and adjuvants, and routes of sensitization. More in-depth analysis of specific strains followed by functional evaluation using microbiota transfer and screening of fecal metabolites is needed for definitive conclusions. At the very least, however, we believe that enrichment for microbiota members *Verrucomicrobia*, *Bacteroidales*, and *Caproiciproducens* in BNP-treated mice contributes to sustained suppression of allergy even in the setting of repeated peanut exposure.

A theme that has emerged from our data is that sustained lowering of allergic disease status is potentially benefitted by gut microbiota enriched for bacteria with known benefits for metabolic health. The parallel rise of obesity and allergy and their increased prevalence in Western societies hints at a potential relationship (77, 78). The Western diet and urban environment have been implicated in higher rates of both allergy and obesity. In a recent study, Hussain et al. used a murine model of allergy to ovalbumin in conjunction with a high-fat diet to show that mice made obese on a high-fat diet were more susceptible to food allergy and this susceptibility was transferrable *via* gut microbiota to non-obese mice (40). Interestingly, in this study, *Verrucomicrobia* were enriched in the gut of obese allergic mice but were decreased in the recipient mice that were rendered allergic post-transfer. Instead, recipient mice showed increased *Lachnospiracea* abundance. Mechanistic understanding of the interrelationships between allergy and metabolic status is currently a subject of intense investigation. Key pathways that intersect the fields of metabolism and immunity implicate a pivotal role in fatty acid utilization and mTOR signaling, which is a nutrient-sensing pathway (79–81). As benefits of OIT alone on protection from anaphylaxis were transient, the loss of therapeutic effects was just a few weeks after stopping OIT (82). Our previous study suggested that reactions in the OIT alone group were extremely severe including loss of mice due to death from anaphylaxis (20), it was prudent at the time to not subject Sham, BBR, and AS groups to OIT. Though allergen challenge may disrupt the gut microbiome shortly after the challenge. We collected fecal samples 8 weeks after the final oral challenge. We believe this time period is sufficient for regaining the stable status of the gut microbiome and represents a time point that provides information regarding lasting changes. Nevertheless, limitations in this study lie in no boiled PN OIT alone group and only BBR + AS group, which makes data interpretation more complicated. Further research into how BNP-induced shift in gut microbiota contributes to IgE-reduction, studies of microbiome change at different time points, and the consequence of food allergy protection *via* immunometabolism regulation is needed. Further study is also needed to determine total IgE and other total isotype antibodies (total IgG2a, IgG1, total IgA) as well as other isotypes of peanut-specific antibodies (peanut-specific IgG2a, IgG1, and IgA) at different time points to monitor if there is an association between these antibodies and gut microbiome changes. An additional limitation of this study is that in the third round of treatment, we did not add the boiled peanut to other groups but only the BBR/AS group. Since our goal is to investigate an intervention that will be available for individuals with established peanut allergy, we tested in mice if their therapeutic effect such as reduced peanut-specific IgE has been established, adding boiled peanut would not alter the established effect. Since neither the AS nor BBR group showed any therapeutic effect, we did not add boiled peanut to those groups. Our previous study should that boiled peanut was not able to sufficiently sensitize mice compared to roasted peanut (Srivastava et al. unpublished data). Our hypothesis is that adding boiled peanut would not interfere with the BBR/AS-established IgE reduction. This hypothesis is consistent with the finding of a previous publication that boiled peanuts reduced the capability to induce allergic responses in mice (83). Our data showed that adding the third component of the treatment regimen i.e. boiled peanut did not alter the BBR/AS-established IgE-reduction effect. For the same reason, since neither BBR nor AS alone group showed any therapeutic effect compared to Sham-treated peanut allergic mice, we did not pursue an additional course of treatment to those control groups to BBR and AS alone groups. However, in future research design, we should add boiled peanut to other control groups and compare with both testing and control groups that are not adding boiled peanut for comparison to provide additional evidence to support our hypothesis (83). We should also add additional treatment courses to BBR and AS alone groups even if they had not shown therapeutic effects to learn that additional courses of treatment may not have significant changes.

In summary, we found that oral therapy with natural medicines containing BBR induced profound and lasting reduction of IgE and IgE-producing B cells leading to tolerance of peanut in peanut-allergic mice. Distinct microbiota profiles were observed in peanut-allergic mice and those rendered tolerant after treatment with BNP. Identified bacterial taxa in this study with known action to increase intestinal epithelial integrity were strongly and inversely correlated with post-challenge plasma histamine and specific IgE. This study provides insight into important biological markers of food allergy for future mechanistic and therapeutic investigation.

## Data availability statement

The original contributions presented in the study are publicly available. This data can be found here: 10.6084/m9.figshare.23708382.

## Ethics statement

The animal study was reviewed and approved by Mount Sinai vivarium.

## Author contributions

KS, OF, NY and YS performed the experiment and data analyses. MC contributed to analysis and manuscript preparation. MM, AN-W and HS revised the manuscript. JZ and X-ML funded the study, and X-ML contributed to the conception of the study. All authors contributed to the article and approved the submitted version.
